# *Colletotrichum* Species Causing Anthracnose in Ipê Trees

**DOI:** 10.3390/jof12040284

**Published:** 2026-04-17

**Authors:** Elder F. M. Silva, Ana G. G. Amaral, André N. Oliveira, Luis O. Viteri, Cristiano B. Moraes, Eugênio E. Oliveira, Ailton Reis, Lavínia G. A. Freitas, Gil R. Santos, Marcos P. S. Câmara

**Affiliations:** 1 Departamento de Agronomia, Universidade Federal Rural de Pernambuco, Recife 52171-900, PE, Brazil; elderfelipe16@gmail.com (E.F.M.S.); andrenunes2110@gmail.com (A.N.O.); lavinia.gafreitas@ufrpe.br (L.G.A.F.); 2 Instituto Federal de Educação, Ciência e Tecnologia do Sertão Pernambucano, Campus Floresta, Floresta 56400-000, PE, Brazil; gabriele.160713@gmail.com; 3 Programa de Pós-Graduação em Produção Vegetal, Universidade Federal do Tocantins, Gurupi 77402-970, TO, Brazil; luis.viteri@uft.edu.br; 4 Programa de Pós-Graduação em Biotecnologia, Universidade Federal do Tocantins, Gurupi 77402-970, TO, Brazil; eugenio@ufv.br; 5 Programa de Pós-Graduação em Ciências Florestais e Ambientais, Universidade Federal do Tocantins, Gurupi 77402-970, TO, Brazil; cbmoraes@mail.uft.edu.br; 6 Departamento de Entomologia, Universidade Federal de Federal de Viçosa, Viçosa 36570-900, MG, Brazil; 7 Departamento de Fitopatologia, Embrapa Hortaliças, Brasília 70359-970, DF, Brazil; ailton@cnph.embrapa.br

**Keywords:** leaf disease, phylogeny, *Handroanthus* L.

## Abstract

Ipê trees (Bignoniaceae), mainly belonging to the genus *Handroanthus*, are widely used in urban landscaping and reforestation programs in Brazil. Anthracnose, typically associated with species of *Colletotrichum*, represents one of the major diseases affecting ipê seedlings and ornamental trees. However, the etiological agents involved have not yet been fully clarified using modern phylogenetic tools. In this study, we identified *Colletotrichum* species associated with anthracnose in ipê trees from Pernambuco, Brazil. A total of 22 isolates were obtained from symptomatic leaves of *Handroanthus impetiginosus* and *H. chrysotrichus*. Species identification was based on multilocus phylogenetic analyses using CAL, GAPDH, GS, and TUB2 loci. The isolates were assigned to three species: *Colletotrichum siamense*, *C. tropicale*, and *C. karsti*. *Colletotrichum siamense* was the most prevalent species (50%), followed by *C. tropicale* (36.3%), while *C. karsti* represented 13.7% of the isolates. Pathogenicity tests confirmed that all isolates were pathogenic to both ipê species, producing typical anthracnose symptoms. Aggressiveness differed between hosts, with *H. impetiginosus* showing higher susceptibility, as indicated by larger lesion development, whereas *H. chrysotrichus* exhibited lower disease aggressiveness. Thus, our findings represent the first multilocus-based identification of *Colletotrichum* species causing anthracnose in ipê trees, providing new insights into the diversity and epidemiology of this disease in urban environments.

## 1. Introduction

Due to ongoing urbanization driven by population growth, the implementation and improvement of green infrastructure have become increasingly important. These areas contribute significantly to human well-being by offering aesthetic, recreational, educational, and cultural benefits. Ornamental plant species are frequently introduced for this purpose. However, this practice can pose ecological risks, including the intensification of biological invasions, displacement of native species, and biotic homogenization [1,2,3]. Therefore, the use of native plants should be prioritized, as it enriches landscape design, conserves local flora, fosters regional identity, and promotes symbiosis between native flora and fauna [4]. This is particularly relevant in Brazil, a megadiverse country that hosts more plant species than any other nation in the world.

Among the many plant species with ornamental potential in Brazilian cities, the ipê trees of the genus *Handroanthus* are particularly prominent. The genus *Handroanthus*, in the family Bignoniaceae, comprises tree species native to Brazil that can reach 20–35 m in height. Among them, the pink and yellow ipês are particularly notable. In addition to their ornamental value, these trees have economic and pharmacological importance: their wood is used in the furniture industry, their bark has medicinal properties, and their extracts are sources for the development of biorational pesticides [5]. Visually, their canopy provides exceptional aesthetic appeal, making them highly valued in landscape design, especially during peak flowering [6,7]. Furthermore, ipê trees are widely used in urban afforestation due to their striking flowering and morphological diversity, which allows their use in different urban settings [8]. They are currently found across several Brazilian biomes, including the Amazon, Caatinga, Cerrado, Atlantic Forest, and Pantanal [9]. Additionally, they are widely produced in nurseries as native species for use in urban landscaping, reforestation initiatives, and the recovery of degraded forest areas [6].

One of the main limiting factors in the production of ornamental plant seedlings is plant disease, particularly anthracnose, caused by fungi of the genus *Colletotrichum* [10,11,12]. The genus *Colletotrichum* comprises a highly diverse group of fungi, with hundreds of species described worldwide, many of which are associated with important plant diseases in agricultural and forest systems. Recent taxonomic advances, particularly those based on multilocus phylogenetic approaches, have substantially increased the number of recognized species within the genus [13,14].

In Brazil, a considerable diversity of *Colletotrichum* species has been reported to infect a wide range of hosts, especially in tropical and subtropical regions. This diversity continues to expand as new studies employing molecular tools reveal cryptic species and refine species boundaries within species complexes. This genus is among the most important in agronomy due to its broad host range, which includes fruits [15], tuberous roots [16], legumes [17], industrial trees [18], and ornamentals [19]. Consequently, anthracnose is considered one of the most significant diseases affecting both commercial nurseries [20,21] and trees in urban green areas [19]. Infected plants typically exhibit irregular necrotic lesions distributed across the leaf blade. In young plants, the disease can damage the apical bud, causing the symptom known as “tip dieback” [16]. As the disease progresses, lesions coalesce, increasing the affected area and reducing the photosynthetic surface. This leads to leaf necrosis, drying, and eventual defoliation of seedlings [22,23]. Such phytosanitary problems, including those caused by anthracnose, can limit agricultural and industrial productivity and reduce the ornamental value of affected species. As a result, *Colletotrichum* has become one of the most intensively studied fungal genera in the past decade [24].

Cases of anthracnose in ipê trees have been reported across the Americas, with causal agents traditionally identified based on morphological characteristics, such as reproductive structures and colony features (e.g., color and appearance). Affected species belong to the genera *Handroanthus* and *Tabebuia*, including *Handroanthus impetiginosus* (pink ipê), *H. chrysotrichus* (yellow ipê), *Tabebuia billbergii* (purple ipê), and *T. rosea* (pink ipê) [25,26]. Previous studies relying solely on morphology identified the causal agents of leaf spots as *Colletotrichum gloeosporioides* and *Colletotrichum* sp. In contrast, García Seminario [27] reported *Colletotrichum boninense* based exclusively on the ITS region. However, this marker alone does not provide sufficient resolution to accurately distinguish species within the genus *Colletotrichum*, limiting reliable identification, which is essential for taxonomic studies. The use of appropriate multilocus molecular markers significantly improves the accuracy of species delimitation and enhances understanding of the diversity within this group of fungi [28]. In this context, clarifying the etiology of the disease is essential to support more effective control strategies. Therefore, this study aimed to identify the causal agents of anthracnose in yellow and pink ipê trees in the state of Pernambuco.

## 2. Materials and Methods

### 2.1. Sampling and Fungal Isolation

Plant samples were obtained from plants exhibiting characteristic anthracnose symptoms in urban afforestation areas within the Metropolitan Region of Recife (specifically the cities of Recife, Paulista, and Olinda) from March to September 2024. Samples were collected from public squares containing both yellow and pink ipê trees. Lesion fragments were surface-disinfected in 70% ethanol for 30 s, followed by immersion in 1.5% sodium hypochlorite for 2 min, and then rinsed twice with sterile distilled water. Tissue fragments were transferred to Petri dishes containing potato dextrose agar (PDA) (200 g potato, 20 g dextrose, and 20 g agar per 1 L of distilled water) [Merck KGaA, Darmstadt, Germany]. The plates were incubated for seven days at 25 ± 2 °C under a 12 h photoperiod with fluorescent white light. Isolates showing characteristics consistent with the genus *Colletotrichum* (Sutton, 1980) were subcultured to obtain pure cultures, preserved using the Castellani method [29], and deposited in the fungal collection of the Laboratório de Fungos Fitopatogênicos, Universidade Federal Rural de Pernambuco.

### 2.2. DNA Extraction, PCR, and Sequencing

The isolates were grown on a potato dextrose agar (PDA) medium under a 12 h photoperiod for seven days. After incubation, mycelial fragments were obtained from the colony surface for DNA extraction, which was carried out using the cetyltrimethylammonium bromide (CTAB) protocol with minor modifications [30]. A partial region of the glyceraldehyde-3-phosphate dehydrogenase (*GAPDH*) gene was amplified to preliminarily determine the *Colletotrichum* species complex to which the isolates belong and to assess haplotype diversity. The resulting sequences were compared with those in the NCBI database using BLAST (https://blast.ncbi.nlm.nih.gov, accessed on 13 January 2026) for initial species identification. Haplotype diversity was calculated using DnaSP v5 [31]. Two representative isolates per haplotype were randomly chosen for multilocus analysis and subsequent assays. For this analysis, additional loci were amplified, including calmodulin (CAL), glutamine synthetase (GS), and β-tubulin (TUB2). These markers are among the most informative for identifying *Colletotrichum* species across different species complexes [28].

The GAPDH region was amplified and sequenced with the primers with GDF and GDR [32]; CAL with CL1C and CL2C [33]; GAPDH with GDF and GDR [32]; GS with GS-64F and GS-967R [34]; and TUB2 with T1 and Bt2B [35,36]. PCR amplifications were performed in a 25 µL volume reaction containing 12.5 µL of PCR-grade water, 2 µL of template DNA (2.5 ng µL^−1^), 2 µL of each primer (10 µM), 1.25 µL of dimethyl sulfoxide, 2.5 µL of dNTPs (10 mM), 2.5 µL of PCR Buffer and 0.3 µL of Taq DNA polymerase (1 U).

Polymerase chain reactions (PCRs) were performed using the following cycling parameters: CAL—initial denaturation for 5 min at 95 °C, followed by 40 cycles of 95 °C for 30 s, 57 °C for 45 s and 72 °C for 1 min, followed by a final extension at 72 °C for 10 min; GS—initial denaturation for 5 min at 95 °C, followed by 40 cycles of 95 °C for 30 s, 56 °C for 45 s and 72 °C for 1 min, followed by a final extension at 72 °C for 10 min; GAPDH—initial denaturation for 5 min at 95 °C, followed by 40 cycles of 95 °C for 30 s, 60 °C for 45 s and 72 °C for 30 s, followed by a final extension at 72 °C for 10 min; and TUB2—initial denaturation for 5 min at 95 °C, followed by 40 cycles of 95 °C for 30 s, 53 °C for 1 min and 72 °C for 1 min, followed by a final extension at 72 °C for 10 min.

PCR products were purified by ethanol and ammonium acetate precipitation. Sequencing of the selected loci was carried out using the ABI PRISM^®^ BigDye^®^ Terminator v3 Cycle Sequencing Kit (Applied Biosystems, Foster City, CA, USA) on the LABCEN/CCB platform at the Universidade Federal de Pernambuco (UFPE, Recife, Brazil).

### 2.3. Phylogenetic Analyses

Nucleotide sequences, as well as consensus construction, were visually inspected and analyzed using the Staden Package v.2.0.0 [37]. Sequence alignments for each locus were performed using the MAFFT server (https://mafft.cbrc.jp/alignment/server/large.html, accessed on 13 January 2026) [38,39] and manually adjusted, when necessary, in MEGA v.7 [40]. The ex-type sequences of the isolates and reference sequences of *Colletotrichum* from previous studies were retrieved from GenBank and included in the phylogenetic analyses (Table S1). For multilocus analysis, the loci were concatenated using Sequence Matrix v. 1.8 [41]. Phylogenetic trees for individual loci and the combined dataset were reconstructed using the maximum likelihood (ML) approach. Analyses were carried out in IQ-TREE v. 2.1.2 [42], with identical sequences retained in the alignment. The best ML tree was inferred using locus-specific substitution models. Model parameters were estimated independently for each partition with ModelFinder [43,44], allowing different evolutionary rates among partitions (-m MFP -p) in IQ-TREE v. 2.1.2.

#### 2.3.1. Species Recognition

As a criterion for phylogenetic species recognition, the Genealogical Concordance Phylogenetic Species Recognition (GCPSR) method by Dettman et al. [45,46] was applied. Based on GCPSR, a clade is considered an independent lineage if it meets at least one of two sub-criteria: a clade satisfies the concordance criterion when it is present in the majority of individual gene trees or it satisfies the non-discordance criterion when it is strongly supported in the individual tree of at least one gene, without being contradicted by the tree of any other individual gene at the same support level.

#### 2.3.2. Prevalence of *Colletotrichum* Species

The prevalence of *Colletotrichum* species associated with anthracnose in ipê was calculated using the formula of P (%) = (Nx/Nt) × 100, where P represents prevalence (%), Nx corresponds to the number of isolates of a given species, and Nt is the total number of isolates.

#### 2.3.3. Pathogenicity and Aggressiveness Assay

Pathogenicity assays were conducted on healthy leaves of seedlings of *H. impetiginosus* and *H. chrysotrichus.* Leaves were washed in running water. The inoculation was conducted using 5 mm diameter mycelial agar plugs placed at inoculation points previously created by slight abrasion of the leaf surface. The negative control consisted of leaves inoculated with 20 µL of sterile distilled water. Seedlings were maintained in a humid chamber for 48 h, under partial shade at ambient temperature around 25 °C. After 48 h, the humid chamber was removed, and plants were kept under the same environmental conditions. Pathogenicity and aggressiveness were evaluated 7 and 15 days after inoculation. Pathogenicity was evaluated based on the appearance of symptoms, and reisolation was performed to confirm the pathogen and thus fulfill Koch’s postulates. Aggressiveness was assessed by measuring the orthogonal diameter of lesions. The experiment was conducted with two replicates, with each replicate represented by three plants with nine inoculated leaflets. The experiment was repeated twice for all isolates.

#### 2.3.4. Data Analysis

Aggressiveness data were subjected to one-way analysis of variance (ANOVA). When significant differences were detected, means were compared using Tukey’s test at a 5% probability level (*p* ≤ 0.05). All statistical analyses were performed using Statistix 10 software [47].

## 3. Results

### 3.1. Sampling and Fungal Isolation from Three Ipês Handroanthus sp.

**Figure 1:**
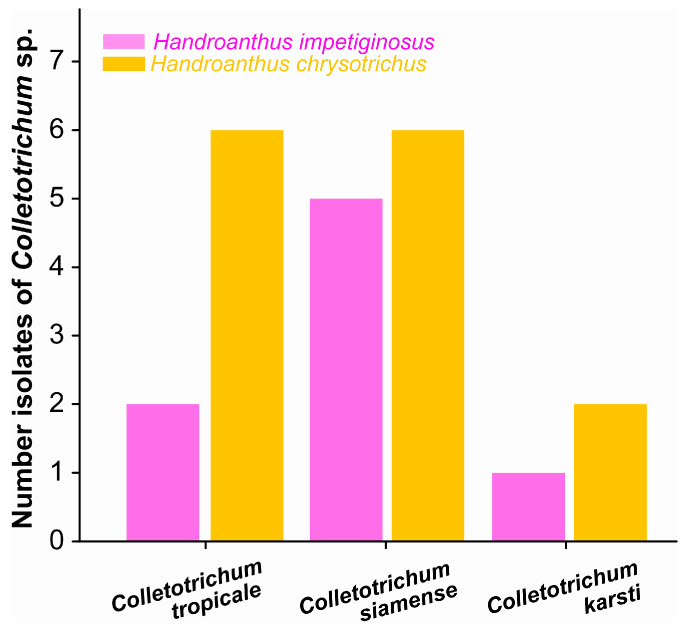
Number of *Colletotrichum* species isolated and associated with pink ipê and yellow ipê. Bars represent the number of isolates of *C. tropicale*, *C. siamense*, and *C. karsti* recovered from each host species.

Analysis of the GAPDH region revealed four haplotypes among *Colletotrichum* isolates obtained from ipê leaves. BLAST comparisons indicated that three haplotypes were highly similar to species within the *C. gloeosporioides* species complex, whereas one haplotype was related to a species from the *C. boninense* complex (Figure 1). The *Colletotrichum siamense* was the most abundant in both ipê species, while *Colletotrichum karsti* was the least abundant.

### 3.2. Phylogenetic Analyses and Species Assignment

**Figure 2:**
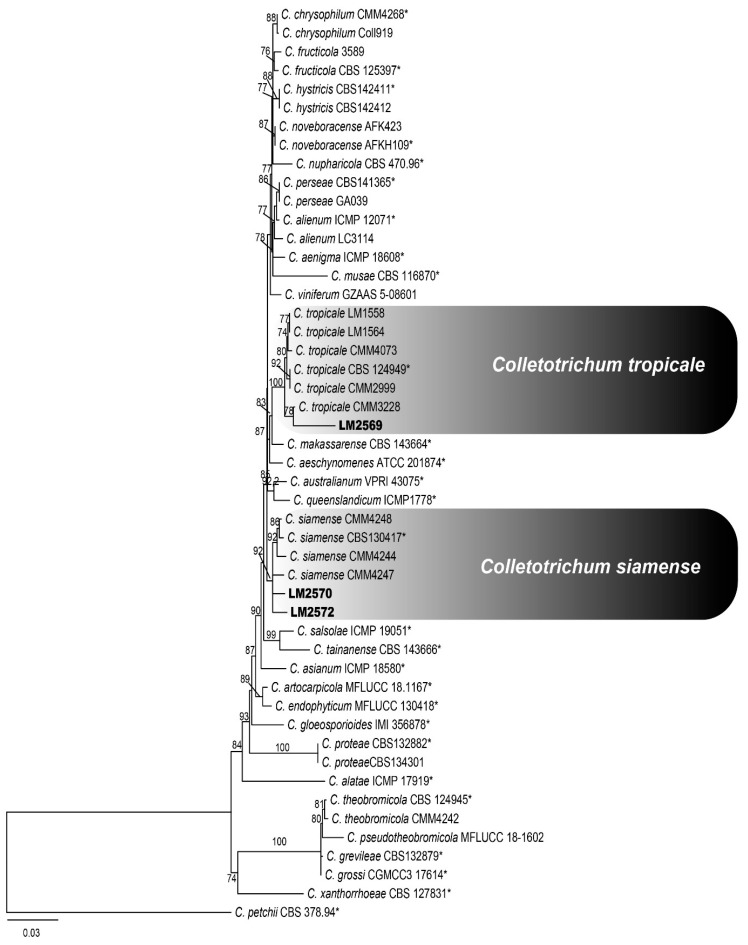
Maximum likelihood tree of the *Colletotrichum gloeosporioides* species complex inferred using IQ-TREE from a concatenated alignment of the GAPDH, TUB2, and GS loci. Significant ML supports (SH-aLRT bootstrap ≥ 80) are indicated. The tree was rooted with *Colletotrichum petchii*. Ex-type isolates are indicated by an asterisk (*) at the end of the taxon labels. Isolates obtained from *Handroanthus* sp. and *Tabebuia* sp. are highlighted in bold.

**Figure 3:**
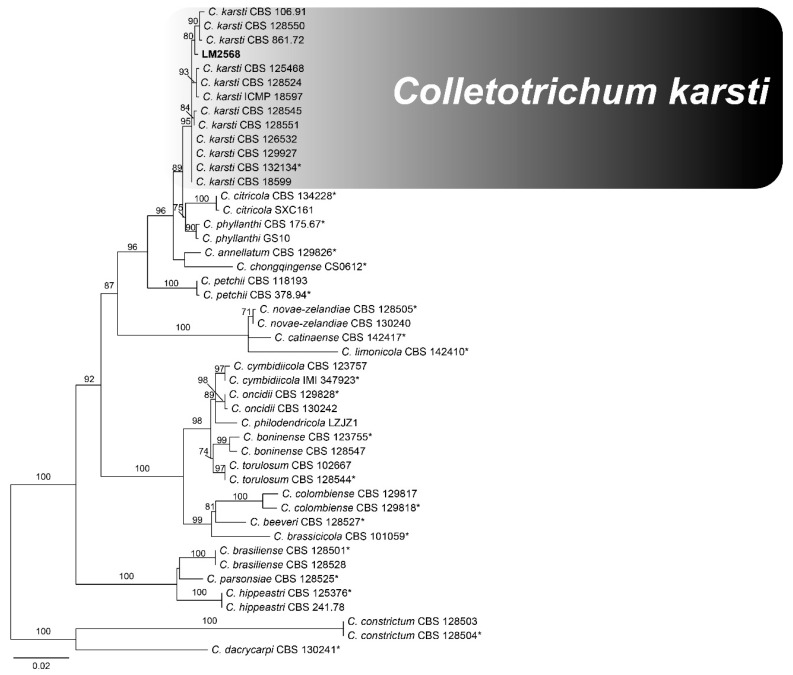
Maximum likelihood tree of the *Colletotrichum* boninense species complex inferred using IQ-TREE from a concatenated alignment of the GAPDH, CAL, and TUB2 loci. Significant ML supports (SH-aLRT bootstrap ≥ 80) are indicated. The tree was rooted with *Colletotrichum dacrycarpi*. Ex-type isolates are indicated by an asterisk (*) at the end of the taxon labels. Isolates obtained from *Handroanthus* sp. and Tabebuia spp. are highlighted in bold.

The GAPDH region revealed a total of four haplotypes among *Colletotrichum* isolates from Ipê leaves. BLAST analysis indicated that three haplotypes showed high similarity with species belonging to the *C. gloeosporioides* species complex, whereas one haplotype showed similarity to a species within the *C. boninense* species complex (Figure 2 and Figure 3).

*Colletotrichum* species assigned to the *C. gloeosporioides* species complex were identified using sequence data from three loci—GS, GAPDH, and TUB2—with the corresponding GenBank accession numbers: GS (PX921655, PX921656, and PX921657), GAPDH (PX921650, PX921651, and PX921652), and TUB2 (PX921653 and PX921654). The second analysis included species assigned to the *C. boninense* species complex. Sequence data from three loci and corresponding GenBank accession numbers—CAL (PX873512), GAPDH (PX873513), and TUB2 (PX873514)—were used for species identification.

### 3.3. Prevalence of Colletotrichum spp. in Handroanthus spp.

**Figure 4:**
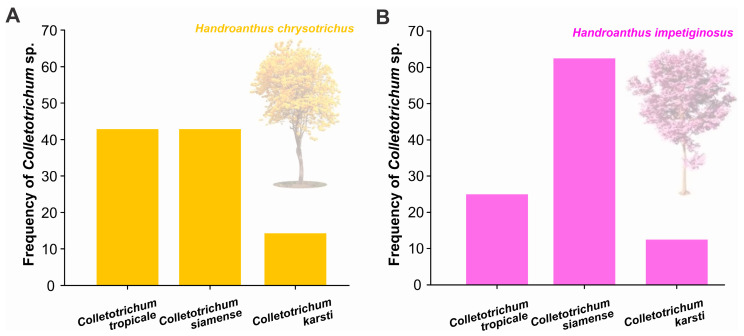
Frequency of *Colletotrichum* species isolated in yellow Ipê (**A**) and pink Ipê (**B**). The stacked bar represents the proportion (%) of *C. tropicale*, *C. siamense*, and *C. karsti* among the total isolates.

The prevalence rates of *Colletotrichum* species causing anthracnose on Ipê trees in Pernambuco were distributed as follows: *Colletotrichum tropicale and C. siamense* (42.86%) and *C. karsti* (14.3%) in yellow ipê *H. chrysotrichus* (Figure 4A). In pink ipê *H. impetiginosus*, the prevalence was 62.5% for *C. siamense*, while for *C. tropicale* and *C. karsti*, prevalence rates were 25% and 12.5%, respectively (Figure 4B). These results indicate broad adaptability and occurrence regardless of host phenotypic variation.

### 3.4. Pathogenicity and Aggressiveness of Colletotrichum spp. in H. chrysotrichus and H. impetiginosus

**Figure 5:**
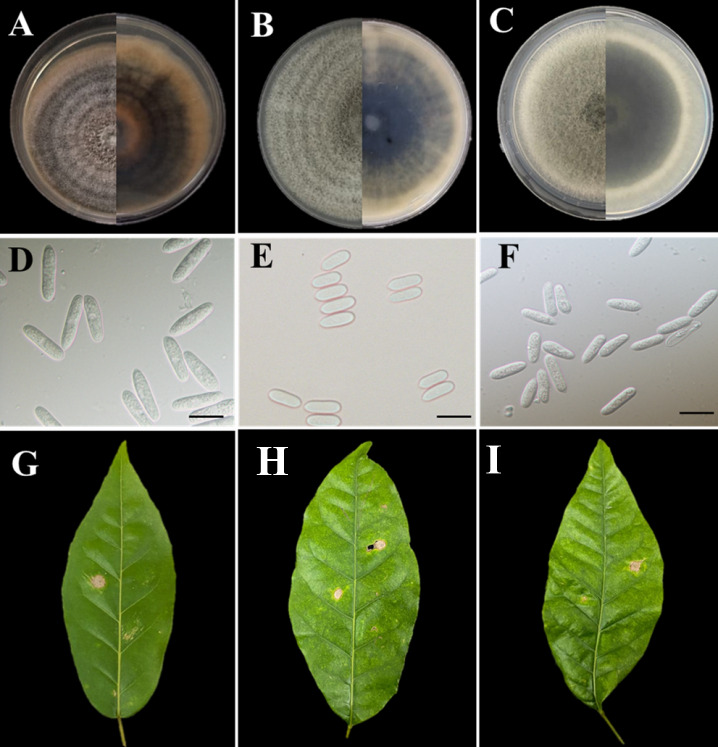
Pathogenicity and aggressiveness of *Colletotrichum* species in *Handroanthus* spp. (**A**,**D**,**G**) *Colletotrichum siamense*. (**B**,**E**,**H**) *Colletotrichum tropicale*. (**C**,**F**,**I**) *Colletotrichum karsti*. (**D**–**F**) Conidia. Scale bars = 10 μm.

**Figure 6:**
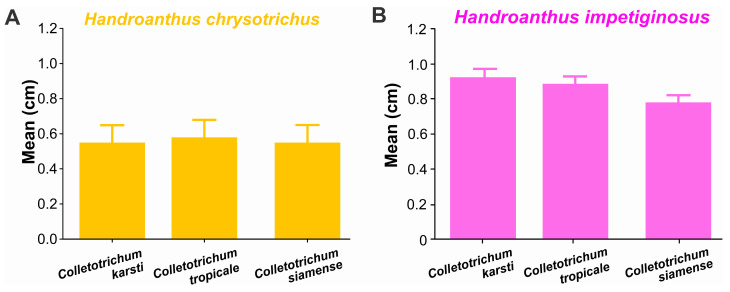
Aggressiveness of *Colletotrichum* species in *Handroanthus chrysotrichus* (**A**) and *Handroanthus impetiginosus* (**B**).

Our pathogenicity test results revealed that all three *Colletotrichum* species were pathogenic, with evident necrotic lesions on *Handroanthus* sp. leaves 48 h after inoculation, in agreement with Koch’s postulates (Figure 5). Similarly, the three *Colletotrichum* species were equally aggressive on both *Handroanthus chrysotrichus* (Figure 6A) and *Handroanthus impetiginosus* (Figure 6B). However, larger *Colletotrichum* lesions were observed on *H. impetiginosus* than on *H. chrysotrichus* (Figure 6).

## 4. Discussion

Here, we provide, for the first time, a multilocus phylogenetic approach to identify *Colletotrichum* species associated with anthracnose in *Handroanthus* species. Our results revealed the presence of two major species complexes, the *C. gloeosporioides* and *C. boninense* complexes, associated with symptomatic ipê leaves in the state of Pernambuco, Brazil. Within these complexes, three species were identified: *C. siamense*, *C. tropicale*, and *C. karsti*. Although all were pathogenic in both *Handroanthus* species, greater aggressiveness was recorded in *H. chrysotrichus* than in *H. impetiginosus*.

The occurrence of multiple *Colletotrichum* species infecting a single host reflects the high taxonomic diversity within the genus and has been frequently reported in studies involving plant diseases caused by this group of fungi [24,48]. Previous studies reporting anthracnose in *Handroanthus* species generally identified the causal agent only as *Colletotrichum* sp. or *C. gloeosporioides*, based mainly on morphological characteristics or on the ITS region [27]. However, the pleomorphic nature of *Colletotrichum* species and the limited phylogenetic resolution of ITS often restrict reliable identification to the genus or species complex level [49,50]. Consequently, multilocus phylogenetic approaches using informative genetic markers have become essential for accurate species delimitation within this genus [28].

Here, we identified species through multilocus phylogenetic analyses of the GAPDH, CAL, GS, and TUB2 loci, which are widely recognized as informative markers for resolving species boundaries in *Colletotrichum* [28,34]. The use of multiple gene regions increases phylogenetic resolution and provides a more robust framework for species recognition compared with single-locus analyses. Similar multilocus approaches have been successfully applied in previous studies investigating the diversity of *Colletotrichum* species associated with several hosts in tropical regions [51,52]. Among the species identified in this study, *C. siamense* was the most prevalent, followed by *C. tropicale*, while *C. karsti* occurred at a lower frequency. The predominance of species within the *C. gloeosporioides* species complex agrees with previous studies showing that this complex comprises some of the most widespread and ecologically versatile species in the genus [28,33]. Members of this complex are known to infect a wide range of hosts and are frequently associated with anthracnose diseases in tropical and subtropical environments.

The pathogenicity assays demonstrated that all isolates could induce typical anthracnose symptoms on both evaluated ipê species. Although no significant differences in aggressiveness were observed among the identified *Colletotrichum* species, differences in susceptibility were detected between the host species. *Handroanthus impetiginosus* showed greater susceptibility than *H. chrysotrichus*, indicating possible differences in host–pathogen interactions. Variations in host susceptibility have also been reported in other pathosystems involving *Colletotrichum* species and may influence disease severity and epidemiological dynamics [51].

The coexistence of multiple pathogenic species associated with anthracnose in ipê trees may have important epidemiological implications. The presence of different *Colletotrichum* species in the same environment can increase the diversity of inoculum sources and potentially favor disease persistence and dissemination. Similar patterns of species coexistence have been documented in other plant hosts, where multiple species contribute to complex disease dynamics [34,52]. Interestingly, some of the species identified in our investigations have also been reported to infect other members of the Bignoniaceae family. For instance, *C. siamense* and *C. karsti* have been reported to cause anthracnose in *Radermachera sinica*, another species within this plant family, suggesting that these pathogens may exhibit a certain degree of host association within Bignoniaceae [53]. The high prevalence and wide geographic distribution of *C. siamense* further support its role as an important pathogen in several plant pathosystems.

## 5. Conclusions

Overall, the identification of *C. siamense*, *C. tropicale*, and *C. karsti* as causal agents of anthracnose in *Handroanthus* species offers new insights into the diversity of pathogens associated with this disease. These results improve the understanding of the etiology and epidemiology of anthracnose in ipê trees and provide a basis for future research on pathogen ecology and disease management in urban forestry systems.

## Data Availability

The original contributions presented in this study are included in the article/Supplementary Material. Further inquiries can be directed to the corresponding authors.

## Supplementary Materials

The following supporting information can be downloaded at https://www.mdpi.com/article/10.3390/jof12040284/s1: Table S1: Collection details and GenBank accession numbers of isolates included in this study.

## Abbreviations

The following abbreviations are used in this manuscript:

PDA	Potato Dextrose Agar
CTAB	Cetyltrimethylammonium Bromide
GAPDH	Glyceraldehyde-3-Phosphate Dehydrogenase
CAL	Calmodulin
GS	Glutamine Synthetase
UFPE	Universidade Federal de Pernambuco
GCPSR	Concordance Phylogenetic Species Recognition
