# Supplementary material for: Colletotrichum Species Causing Anthracnose in Ipê Trees
Source: J Fungi (Basel). 2026 Apr 17;12(4):284. doi: 10.3390/jof12040284 (PMC13117155; doi:10.3390/jof12040284)
Supplement: Supplementary file 1 [file jof-12-00284-s001.zip › jof-4243855-supplementary.pdf]

Table S1. Collection details and GenBank accession numbers of isolates included in this study.

Species complex	species	Collection n°	Host	Country	GenBank accession numbers			
					GAPDH	CAL	TUB	GS
Gloeosporioides	***C.siamense***	**LM2570**	*Handoanthus albus*	Brazil	**PX921651**		**PX921655**	**PX921655**
Gloeosporioides	*C. aenigma*	ICMP 18608*	*Persea americana*	Israel	JX010044	JX010078		JX010389
Gloeosporioides	*C. aeschynomenes*	ATCC 201874*	*Aeschynomene virginica*	USA	JX009930	JX010081		JX010392
Gloeosporioides	*C. alatae*	ICMP 17919*	*Dioscorea alata*	India	JX009990	JX010065		JX010383
Gloeosporioides	*C. alienum*	ICMP 12071*	*Malus domestica*	New Zealand	JX010028	JX010101		JX010411
Gloeosporioides	*C. alienum*	LC3114	*Camellia sinensis*	China	KJ954832	KJ954982		KJ955279
Boninense	*C. annellatum CBS 129826*	CBS 129826	*Hevea brasiliensis*	Colombia	JQ005309	JQ005743	JQ005656	
Gloeosporioides	*C. artocarpicola*	MFLUCC 18-1167*	*Artocarpus heterophyllus*	Thailand	MN435568	-		MN435567
Gloeosporioides	*C. asianum*	HKUCC 10862, ICMP 18605	*Mangifera indica*	Thailand	JX010021	-		-
Gloeosporioides	*C. asianum*	ICMP 18580*, B26CBS 130418	*Coffea arabica*	Thailand	JX010053	JX010096		JX010406
Gloeosporioides	*C. australianum*	VPRI 43075*	*Citrus sinensis*	Australia	MG572127	MG572160		MG572149
Boninense	*C. beeveri CBS 128527*	CBS 128527*	*Brachyglottis repanda*	New Zealand	JQ005258	JQ005692	JQ005605	
Boninense	*C. boninense CBS 123755*	CBS 123755*	*Crinum asiaticum var. sinicum*	Japan	JQ005240	JQ005674	JQ005588	
Boninense	*C. boninense CBS 128547*	CBS 128547	*Camellia sp*	New Zealand	JQ005246	JQ005680	JQ005593	
Boninense	*C. brasiliense CBS 128501*	CBS 128501*	*Passiflora edulis*	Brazil	JQ005322	JQ005756	JQ005669	
Boninense	*C. brasiliense CBS 128528*	CBS 128528	*Passiflora edulis*	Brazil	JQ005321	JQ005755	JQ005668	
Boninense	*C. brassicicola CBS 101059*	CBS 101059*	*Brassica oleracea var. gemmifera*	New Zealand	JQ005259	JQ005693	JQ005606	

Boninense	*C. brassicicola CBS 101059*							
Boninense	*C. camelliae-japonicae CGMCC3.18118*							
Boninense	*C. catinaense CBS 142417*	CBS 142417	*Citrus reticulata*	Italy	KY856224	KY856053	KY856482	
Boninense	*C. chongqingense CS0612*	CS0612	*Camellia sinensis*	China	MG602022	MT976097	MG602044	
Gloeosporioides	*C. chrysophilum*	CMM4268*	*Musa sp.*	Brazil	KX094183	KX094204		KX094285
Gloeosporioides	*C. chrysophilum*	Coll919	*Terpsichore taxifolia*	Puerto Rico	KX094177	KX094207		KX094288
Boninense	*C. citricola CBS 134228*	CBS 134228*	*Citrus unchiu*	China	KC293736	KC293696	KC293656	
Boninense	*C. citricola SXC161*	SXC161		China	KC293738	KC293698	KC293658	
Boninense	*C. colombiense CBS 129817*	CBS 129817	*Passiflora edulis*	Colombia	JQ005260	JQ005694	JQ005607	
Boninense	*C. colombiense CBS 129818*	CBS 129818*	*Passiflora edulis*	Colombia	JQ005261	JQ005695	JQ005608	
Boninense	*C. constrictum CBS 128503*	CBS 128503	*Solanum betaceum*	New Zealand	JQ005324	JQ005758	JQ005671	
Boninense	*C. constrictum CBS 128504*	CBS 128504*	*Citrus limon*	New Zealand	JQ005325	JQ005759	JQ005672	
Boninense	*C. cymbidiicola CBS 123757*	CBS 123757	*Cymbidium sp.*	Japan	JQ005255	JQ005689	JQ005602	
Boninense	*C. cymbidiicola IMI 347923*	IMI 347923*	*Cymbidium sp.*	Australia	JQ005253	JQ005687	JQ005600	
Boninense	*C. dacrycarpi CBS 130241*	CBS 130241*	*Dacrycarpus dacrydioides*	New Zealand	JQ005323	JQ005757	JQ005670	
Boninense	*C. doitungense MFLU14-0128*							
Gloeosporioides	*C. endophyticum*	MFLUCC 130418	*Pennisetum purpureum*	Thailand	KC832854	-		-
Boninense	*C. feijoicola CBS 144633*							
Gloeosporioides	*C. fructicola*	CBS 125397*	*Tetragastris panamensis*	Panama	JX010032	JX010099		JX010409
Gloeosporioides	*C. fructicola*	3589	*Theobroma cacao*	Panama	KX094175	KX094199		KX094280
Boninense	*C. gloeosporioides*	IMI 356878*	*Citrus sinensi*	Italy	JX010056	JX010085		JX010445
Gloeosporioides	*C. grevileae*	CBS132879	*Grevillea sp.*	taly	KC297010	KC297033		KC297102
Gloeosporioides	*C. grossum*	CGMCC 3.17614, CAUG7,	*Chili pepper*	China	KP890159	-		KP890171

		LC6227*						
Boninense	*C. hippeastri CBS 125376*	CBS 125376*	*Hippeastrum vittatum*	China	JQ005318	JQ005752	JQ005665	
Boninense	*C. hippeastri CBS 241.78*	CBS 241.78	*Hippeastrum sp.*	Netherlands	JQ005319	JQ005753	JQ005666	
Gloeosporioides	*C. hystricis*	CBS142411*	*Citrus hystrix*	Italy	KY856274	-		KY856532
Gloeosporioides	*C. hystricis*	CBS142412	*Citrus hystrix*	Italy	KY856275	-		KY856533
Boninense	*C. karstii CBS 106.91*	CBS 106.91	*Carica papaya*	Brazil	JQ005307	JQ005741	JQ005654	
Boninense	*C. karstii CBS 125468*	CBS 125468	*Coffea* sp.,	vietman	JQ005284	JQ005718	JQ005631	
Boninense	*C. karstii CBS 126532*	CBS 126532	*Citrus* sp.	south africa	JQ005296	JQ005730	JQ005643	
Boninense	*C. karstii CBS 128524*	CBS 128524	*Citrullus lanatus*	New Zealand	JQ005282	JQ005716	JQ005629	
Boninense	*C. karstii CBS 128545*	CBS 128545	*Capsicum annuum*	New Zealand	JQ005294	JQ005728	JQ005641	
Boninense	*C. karstii CBS 128550*	CBS 128550	*Annona cherimola*	Mexico	JQ005306	JQ005740	JQ005653	
Boninense	*C. karstii CBS 128551*	CBS 128551	*Citrus* sp.	New Zealand	JQ005295	JQ005729	JQ005642	
Boninense	*C. karstii CBS 129927*	CBS 129927	*Anthurium* sp.	Thailand	JQ005293	JQ005727	JQ005640	
Boninense	*C. karstii CBS 132134*	CBS 132134*	*Vanda sp.*	China	HM585391	HM582013	HM585428	
Boninense	*C. karstii CBS 861.72*	CBS 861.72	*Bombax aquaticum*	Brazil	JQ005271	JQ005705	JQ005618	
Boninense	*C. karstii ICMP 18597*	ICMP 18597	*Clivia miniata*	Japan	JQ005283	JQ005717	JQ005630	
Boninense	*C. karstii ICMP 18599*	CBS 18599	*Cucumis melo*	Japan	JQ005278	JQ005712	JQ005625	
Boninense	*C. limonicola CBS 142410*	CBS 142410	*Citrus limon*	Malta	KY856296	KY856125	KY856554	
Gloeosporioides	*C. makassarense*	CBS 143664*	*Capsicum annuum*	Indonesia	MH728820	MH748264		MH846563
Boninense	*C. musae*	CBS 116870*, ICMP 19119	*Musa sp.*	USA	JX010050	JX010103		HQ596280
Boninense	*C. novae-zelandiae CBS 128505*	CBS 128505*	*Capsicum annuum*	New Zealand	JQ005315	JQ005749	JQ005662	
Boninense	*C. novae-zelandiae CBS 130240*	CBS 130240	*Citrus sp. (grapefruit)*	New Zealand	JQ005316	JQ005750	JQ005663	
Gloeosporioides	*C. noveboracense*	AFKH109*	*Apple/Idared*	Columbia/NY	MN640567	MN640568		MN640569

Gloeosporioides	*C. noveboracense*	AFK423	*Malus domestica*	Ulster/ NY	MN741085	MN741099		MN701194
Gloeosporioides	*C. nupharicola*	CBS 470.96*, ICMP 18187	*Nuphar lutea subsp. polysepala*	USA	JX009936	JX010088		JX010397
Gloeosporioides	*C. nupharicola*	CBS 472.96, ICMP 17940	*Nymphaea odorata*	USA	JX010031	JX010089		JX010399
Boninense	*C. oncidii CBS 129828*	CBS 129828*	*Oncidium sp.*	Germany	JQ005256	JQ005690	JQ005603	
Boninense	*C. oncidii CBS 130242*	CBS 130242	*Oncidium sp.*	Germany	JQ005257	JQ005691	JQ005604	
Boninense	*C. parsonsiae CBS 128525*	CBS 128525*	*Parsonsia capsularis*	New Zealand	JQ005320	JQ005754	JQ005667	
Boninense	*C. parsonsiae CGMCC 3.15126*							
Gloeosporioides	*C. perseae*	GA039	*Persea americana*	Israel	KX620236	KX620269		KX620335
Gloeosporioides	*C. perseae*	CBS141365*	*Persea americana*	Israel	KX620242	KX620275		KX620341
Gloeosporioides	*C. petchii*	CBS 378.94*	*Dracaena marginata*	Italy	JQ005310	-		JQ005657
Boninense	*C. petchii CBS 118193*	CBS 118193	*Dracaena sanderiana*	China	JQ005314	JQ005748	JQ005661	
Boninense	*C. petchii CBS 378.94*	CBS 378.94*	*Dracaena marginata*	Italy	JQ005310	JQ005744	JQ005657	
Boninense	*C. philodendricola LZJZ1*	LZJZ1	*Philodendron tatei cv. Congo*	China	MH105261	MH105281	MH105277	
Gloeosporioides	*C. phyllanthi*	GS10	*B. variegata*	India	JX576718	-		JX576715
Boninense	*C. phyllanthi CBS 175.67*	CBS 175.67*	*Phyllanthus acidus*	India	JQ005308	JQ005742	JQ005655	
Boninense	*C. phyllanthi GS10*	GS10	*Bauhinia variegata*	India	JX576718	JX863678	JX863674	
Gloeosporioides	*C. proteae*	CBS132882*	*Protea sp.*	South Africa	KC297009	KC297032		KC297101
Gloeosporioides	*C. proteae*	CBS134301	*Protea sp.*	South Africa	KC842379	KC842381		KC842387
Gloeosporioides	*C. pseudoboninense*	CBS 123921	*Dendrobium kingianum*	Japan	JQ005250			JQ005597
Boninense	*C. pseudoboninense LZJZ5*							
Gloeosporioides	*C. pseudotheobromicola*	MFLUCC 18-1602	*Prunus avium*	China	MH853675	-		MH853684
Gloeosporioides	*C. queenslandicum*	ICMP1778	*Anacardium occidentale*	Brazil	MF110850	MF110997		MF111060
Gloeosp	*C. salsolae*	ICMP	*Salsola tragus*	Hungar	JX009	JX010		JX010

orioides		19051*		y	916	093		403
Gloeosporioides	*C. siamense*	LM130	*Carica papaya*	Brazil	MN228552	ON063603		-
Boninense	*C. siamense*	ICMP 18578*, CBS 130417	*Coffea arabica*	Thailand	JX009924	-		-
Gloeosporioides	*C. siamense*	CBS130417	*Coffea arabica*	Thailand	-	JX010094		JX010404
Gloeosporioides	*C. siamense*	CMM4247	*Musa sp.*	Brazil	KX094155	KX094196		KX094261
Gloeosporioides	*C. siamense*	CMM4081	*Mangifera indica*	Brazil	KX094166	KX094220		KX094272
Gloeosporioides	*C. siamense*	CMM4244	*Musa sp.*	Brazil	KX094172	KX094226		KX094299
Gloeosporioides	*C. siamense*	CMM4248	*Musa sp.*	Brazil	KX094154	KX094229		KX094300
Gloeosporioides	*C. tainanense*	CBS 143666*	*Capsicum annuum*	Taiwan	MH728823	MH748259		MH846558
Gloeosporioides	*C. theobromicola*	CBS 124945*, ICMP 18649	*Theobroma cacao*	Panama	JX010006	JX010139		JX010447
Gloeosporioides	*C. theobromicola*	CMM4242	*Musa sp.*	Brazil	KX094173	KX094197		KX094278
Gloeosporioides	*C. theobromicola*	GJS0843	*Theobroma cacao*	Panama	-	-		GU994476
Boninense	*C. torulosum CBS 102667*	CBS 102667	*Passiflora edulis*	New Zealand	JQ005252	JQ005686	JQ005599	
Boninense	*C. torulosum CBS 128544*	CBS 128544*	*Solanum melongena*	New Zealand	JQ005251	JQ005685	JQ005598	
Gloeosporioides	*C. tropicale*	CBS 124949*, ICMP 18653	*Theobroma cacao*	Panama	JX010007	JX010097		GU994454
Gloeosporioides	*C. tropicale*	CMM2999	*Anacardium occidentale*	Brazil	MF110846	MF111038		MF111088
Gloeosporioides	*C. tropicale*	CMM3228	*Anacardium occidentale*	Brazil	MF110905	MF111039		-
Gloeosporioides	*C. tropicale*	LM1558	*Anacardium occidentale*	Brazil	PP112352	PP112365		PP112374
Gloeosporioides	*C. tropicale*	LM1564	*Anacardium occidentale*	Brazil	PP112354	PP112366		PP112376
Gloeosporioides	*C. tropicale*	CMM 4073	*Mangifera indica*	Brazil	KC517185	KC430870		KC517262
Gloeosporioides	*C. viniferum*	GZAAS 5.08601*	*Vitis vinifera*	China	JN412798	-		JN412813
Gloeosporioides	*C. xanthorrhoeae*	CBS 127831*	*Xanthorrhoea preissii*	Australia	JX009927	JX010138		JX010448
Boninen	***C.karsti***	**LM2568**	*Handoanthus*	Brazil	**PX873**	**PX87**	**PX87**	

se			*albus*		**512**	**3513**	**3513**	
Gloeosp	***C.tropicale***	**LM2572**	*Handoanthus*		**PX921**		**PX92**	**PX92**
orioides			*albus*	Brazil	**6510**		**1654**	**1656**

CBS: Centraalbureau voor Schimmelcultures, Utrecht, The Netherlands; CMM: Culture Collection of Phythopathogenic Fung "Prof. Maria Menezes", Recife, Brazil; GZAAS: Guizhou Academy of Agricultural Sciences Herbarium, China; ICMP: International Collection of Microorganisms from Plants, Auckland, New Zealand; LC: Working collection of Lei Cai, housed at CAS, China; MFLUCC: Mae Fah Luang University Culture Collection, ChiangRai, Thailand; LM: Working collection of Laboratório de Micologia, housed at UFRPE, Brazil; CGMCC: China General Microbiological Culture Collection Center, China; GZAAS:herbarium of Guizhou Academy of Agricultural Sciences, China; JZB: Culture collections of Beijing Academy of Agricultural and Forestry Sciences, China. * = ex-type culture. Strains collected and sequences generated for this study are bold font. CAL: calmodulin; GAPDH: glyceraldehyde-3-phosphate dehydrogenase; GS: glutamine synthetase; TUB2: β-tubulin
